# Supplementary material for: Second-line systemic treatment for metastatic colorectal cancer: A systematic review and Bayesian network meta-analysis based on RCT
Source: PLoS One. 2024 Dec 23;19(12):e0313278. doi: 10.1371/journal.pone.0313278 (PMC11666018; doi:10.1371/journal.pone.0313278)
Supplement: S1 File — (DOCX) [file pone.0313278.s001.docx]

Table 1: Studies search strategy

(1)

PubMed

(((((((((Colorectal Neoplasms[MeSH Terms]) OR (Colorectal Neoplasms[Title/Abstract]) OR (Colorectal Tumor*[Title/Abstract])) OR (Colorectal Cancer*[Title/Abstract])) OR (Colorectal Carcinoma*[Title/Abstract])) OR (neoplastic colorectal[Title/Abstract])) OR (colorectal neoplas*[Title/Abstract])) OR ((((((((((((((Colonic Neoplasms[MeSH Terms]) OR (Colonic Neoplasm*[Title/Abstract])) OR (Colon Neoplasm*[Title/Abstract])) OR (Colon Cancer*[Title/Abstract])) OR (Colonic Cancer*[Title/Abstract])) OR (Colon Adenocarcinoma*[Title/Abstract])) OR (colon mass* (tumor[Title/Abstract]))) OR (colon neoplasia[Title/Abstract])) OR (colon tumo*[Title/Abstract])) OR (colonic mass* (tumor[Title/Abstract]))) OR (colonic neoplasia[Title/Abstract])) OR (colonic tumo*[Title/Abstract])) OR (mesocolon tumo*[Title/Abstract])) OR (neoplastic colon*[Title/Abstract]))) OR (((((((((Rectal Neoplasms[MeSH Terms]) OR (Rectal Neoplasm*[Title/Abstract])) OR (Rectum Neoplasm*[Title/Abstract])) OR (Rectal Tumor*[Title/Abstract])) OR (Rectum Cancer*[Title/Abstract])) OR (Rectal Cancer*[Title/Abstract])) OR (rectal carcinogenesis[Title/Abstract])) OR (rectal malignanc*[Title/Abstract])) OR (rectum malignancy[Title/Abstract]))) AND (((((((((((((((((((((((((((((fluorouracil[Title/Abstract]) OR (fluoropyrimidine[Title/Abstract])) OR (oxaliplatin[Title/Abstract])) OR (irinotecan[Title/Abstract])) OR (folinic acid[Title/Abstract])) OR (leucovorin[Title/Abstract])) OR (mitomycin-C[Title/Abstract])) OR (raltitrexed[Title/Abstract])) OR (capecitabine[Title/Abstract])) OR (S-1[Title/Abstract])) OR (abituzumab[Title/Abstract])) OR (vatalanib[Title/Abstract])) OR (gefitinib[Title/Abstract])) OR (trebananib[Title/Abstract])) OR (bevacizumab[Title/Abstract])) OR (panitumumab[Title/Abstract])) OR (cetuximab[Title/Abstract])) OR (ramucirumab[Title/Abstract])) OR (conatumumab[Title/Abstract])) OR (ganitumab[Title/Abstract])) OR (aflibercept[Title/Abstract])) OR (regorafenib[Title/Abstract])) OR (linifanib[Title/Abstract])) OR (hyaluronan[Title/Abstract])) OR (dabrafenib[Title/Abstract])) OR (trametinib[Title/Abstract])) OR (trastuzumab[Title/Abstract])) OR (lapatinib[Title/Abstract])) OR (second line[Title/Abstract]))) AND ((((((Random*[Title/Abstract]) OR (RCT[Title/Abstract])) OR (Randomized controlled trial[Title/Abstract])) OR (Cohort[Title/Abstract])) OR (prospective[Title/Abstract])) OR (retrospective[Title/Abstract]))

(2)

EMBASE

#1: 'colorectal tumor'/exp

#2: 'colorectal tumor*':ab,ti OR 'colorectal cancer*':ab,ti OR 'colorectal carcinoma*':ab,ti OR 'neoplastic colorectal':ab,ti OR 'colorectal neoplas*':ab,ti

#3: #1 OR #2

#4: 'colon cancer'/exp

#5: 'colonic neoplasm*':ab,ti OR 'colon neoplasm*':ab,ti OR 'colon cancer*':ab,ti OR 'colonic cancer*':ab,ti OR 'colon adenocarcinoma*':ab,ti OR ('colon mass*':ab,ti AND tumor:ab,ti) OR 'colon neoplasia':ab,ti OR 'colon tumo*':ab,ti OR ('colonic mass*':ab,ti AND tumor:ab,ti) OR 'colonic neoplasia':ab,ti OR 'colonic tumo*':ab,ti OR 'mesocolon tumo*':ab,ti OR 'neoplastic colon*':ab,ti

#6: #4 OR #5

#7: 'rectum cancer'/exp

#8: 'rectal neoplasm*':ab,ti OR 'rectum neoplasm*':ab,ti OR 'rectal tumor*':ab,ti OR 'rectum cancer*':ab,ti OR 'rectal cancer*':ab,ti OR 'rectal carcinogenesis':ab,ti OR 'rectal malignanc*':ab,ti OR 'rectum malignancy':ab,ti

#9: #7 OR #8

#10: #3 OR #6 OR #9

#11: second line':ab,ti OR fluorouracil:ab,ti OR fluoropyrimidine:ab,ti OR oxaliplatin:ab,ti OR irinotecan:ab,ti OR 'folinic acid':ab,ti OR leucovorin:ab,ti OR 'mitomycin c':ab,ti OR raltitrexed:ab,ti OR capecitabine:ab,ti OR 's 1':ab,ti OR abituzumab:ab,ti OR vatalanib:ab,ti OR gefitinib:ab,ti OR trebananib:ab,ti OR bevacizumab:ab,ti OR panitumumab:ab,ti OR cetuximab:ab,ti OR ramucirumab:ab,ti OR conatumumab:ab,ti OR ganitumab:ab,ti OR aflibercept:ab,ti OR regorafenib:ab,ti OR linifanib:ab,ti OR hyaluronan:ab,ti OR dabrafenib:ab,ti OR trametinib:ab,ti OR trastuzumab:ab,ti OR lapatinib:ab,ti

#12: random*:ab,ti OR rct:ab,ti OR 'randomized controlled trial':ab,ti OR cohort:ab,ti OR prospective:ab,ti OR retrospective:ab,ti

#13: #10 AND #11 AND #12

(3)

Web of Science

#1 (((((((((((TI=(Colorectal Neoplasms)) OR AB=(Colorectal Neoplasms)) OR TI=(Colorectal Tumor*)) OR AB=(Colorectal Tumor*)) OR TI=(Colorectal Cancer*)) OR AB=(Colorectal Cancer*)) OR TI=(Colorectal Carcinoma*)) OR AB=(Colorectal Carcinoma*)) OR TI=(neoplastic colorectal)) OR AB=(neoplastic colorectal)) OR TI=(colorectal neoplas*)) OR AB=(colorectal neoplas*)

#2 (((((((((((((((((((((((((((TI=(Colonic Neoplasms)) OR AB=(Colonic Neoplasms)) OR TI=(Colonic Neoplasm*)) OR AB=(Colonic Neoplasm*)) OR TI=(Colon Neoplasm*)) OR AB=(Colon Neoplasm*)) OR TI=(Colon Cancer*)) OR AB=(Colon Cancer*)) OR TI=(Colonic Cancer*)) OR AB=(Colonic Cancer*)) OR TI=(Colon Adenocarcinoma*)) OR AB=(Colon Adenocarcinoma*)) OR TI=(colon mass* (tumor))) OR AB=(colon mass* (tumor))) OR TI=(colon neoplasia)) OR AB=(colon neoplasia)) OR TI=(colon tumo*)) OR AB=(colon tumo*)) OR TI=(colonic mass* (tumor))) OR AB=(colonic mass* (tumor))) OR TI=(colonic neoplasia)) OR AB=(colonic neoplasia)) OR TI=(colonic tumo*)) OR AB=(colonic tumo*)) OR TI=(mesocolon tumo*)) OR AB=(mesocolon tumo*)) OR TI=(neoplastic colon*)) OR AB=(neoplastic colon*)

#3 (((((((((((((((TI=(Rectal Neoplasm*)) OR AB=(Rectal Neoplasm*)) OR TI=(Rectum Neoplasm*)) OR AB=(Rectum Neoplasm*)) OR TI=(Rectal Tumor*)) OR AB=(Rectal Tumor*)) OR TI=(Rectum Cancer*)) OR AB=(Rectum Cancer*)) OR TI=(Rectal Cancer*)) OR AB=(Rectal Cancer*)) OR TI=(rectal carcinogenesis)) OR AB=(rectal carcinogenesis)) OR TI=(rectal malignanc*)) OR AB=(rectal malignanc*)) OR TI=(rectum malignancy)) OR AB=(rectum malignancy)

#4 #1 OR #2 OR #3

#5 (((((((((((((((((((((((((((((((((((((((((((((((((TI=(second line)) OR AB=(second line)) OR TI=(fluorouracil)) OR AB=(fluorouracil)) OR TI=(fluoropyrimidine )) OR AB=(fluoropyrimidine )) OR TI=(oxaliplatin)) OR AB=(oxaliplatin)) OR TI=(irinotecan)) OR AB=(irinotecan)) OR TI=(folinic acid)) OR AB=(folinic acid)) OR TI=(leucovorin)) OR AB=(leucovorin)) OR TI=(mitomycin-C)) OR AB=(mitomycin-C)) OR TI=(raltitrexed)) OR AB=(raltitrexed)) OR TI=(capecitabine)) OR AB=(capecitabine)) OR TI=(S-1)) OR AB=(S-1)) OR TI=(abituzumab)) OR AB=(abituzumab)) OR TI=(vatalanib)) OR AB=(vatalanib)) OR TI=(gefitinib)) OR AB=(gefitinib)) OR TI=(trebananib)) OR AB=(trebananib)) OR TI=(bevacizumab)) OR AB=(bevacizumab)) OR TI=(panitumumab)) OR AB=(panitumumab)) OR TI=(cetuximab)) OR AB=(cetuximab)) OR TI=(ramucirumab)) OR AB=(ramucirumab)) OR TI=(conatumumab)) OR AB=(conatumumab)) OR TI=(ganitumab)) OR AB=(ganitumab)) OR TI=(aflibercept)) OR AB=(aflibercept)) OR TI=(regorafenib)) OR AB=(regorafenib)) OR TI=(linifanib)) OR AB=(linifanib)) OR TI=(hyaluronan)) OR AB=(hyaluronan)

#6 (((((((((((TI=(Random*)) OR AB=(Random*)) OR TI=(RCT)) OR AB=(RCT)) OR TI=(Randomized controlled trial)) OR AB=(Randomized controlled trial)) OR TI=(Cohort)) OR AB=(Cohort)) OR TI=(prospective)) OR AB=(prospective)) OR TI=(retrospective)) OR AB=(retrospective)

#7 #4 AND #5 AND #6

(4)

Cochrane Library

#1 MeSH descriptor: [Rectal Neoplasms] explode all trees 2385

#2 (Rectal Neoplasm* OR Rectum Neoplasm* OR Rectal Tumor* OR Rectum Cancer* OR Rectal Cancer* OR rectal carcinogenesis OR rectal malignanc* OR rectum malignancy):ti,ab,kw 8377

#3 #1 OR #2 8514

#4 MeSH descriptor: [Colorectal Neoplasms] explode all trees 10857

#5 (Colorectal neoplasms OR Colorectal Tumor* OR Colorectal Cancer* OR Colorectal Carcinoma* OR neoplastic colorectal OR colorectal neoplas*):ti,ab,kw 18917

#6 #4 OR #5 21769

#7 MeSH descriptor: [Colonic Neoplasms] explode all trees 2195

#8 (Colonic Neoplasm* OR Colon Neoplasm* OR Colon Cancer* OR Colonic Cancer* OR Colon Adenocarcinoma* OR colon mass* (tumor) OR colon neoplasia OR colon tumo* OR colonic mass* (tumor) OR colonic neoplasia OR colnic tumo* OR mesocolon tumo* OR neoplastic colon*):ti,ab,kw 8978

#9 #7 OR #8 9006

#10 #3 OR #6 OR #9 28358

#11 (fluorouracil OR fluoropyrimidine OR irinotecan OR folinic acid OR leucovorin OR mitomycin-C OR raltitrexed OR capecitabine OR S-1 OR abituzumab OR vatalanib OR gefitinib OR trebananib OR bevacizumab OR panitumumab OR cetuximab OR ramucirumab OR conatumumab OR ganitumab OR aflibercept OR regorafenib OR linifanib OR hyaluronan):ti,ab,kw 35960

#12 (Random* OR RCT OR Randomized controlled trial OR Cohort OR prospective OR retrospective):ti,ab,kw 1267552

#13 #10 AND #11 AND #12 5849

Table 2: Basic characteristics of each studies included in the meta-analysis

| Author | Center | Time | TreatmentA | TreatmentB | Sample size(Male/Female) | | Age | | ECOG status RAS status | | prior treatment |
| --- | --- | --- | --- | --- | --- | --- | --- | --- | --- | --- | --- |
|  |  |  |  |  | Treatment  A | Treatment  B | Treatment  A | TreatmentB | Treatment  A/B |  |  |
| Aparicio 2022^[23]^ | Spain | 2019.2-2020.11 | FOLFIRI+ Panitumumab | FOLFIRI | 18  (10/8） | 13  (10/3) | 59  (51–66) | 67  (62–74) | 0-1/0-1 | WT | FOLFIRI+ Panitumumab |
| Be´couarn 2001^[24]^ | France | 1997.7-1999.4 | FOLFIRI+ FOLFOX | Irinotecan+ Oxaliplatin | 32  (20/12) | 30  (20/10) | 64  (44-75) | 63  (43-76) | ECOG:  0-2/0-2 | NA | 5FU-based |
| Bendell 2013^[25]^ | Multi-  center | 2008.3-2009.8 | FOLFIRI+ Axitinib | FOLFIRI+ Bevacizumab | 49  (31/18) | 51  (27/24) | 59  (24-76) | 58  (34-80) | ECOG:  0-1/0-1 | NA | Oxaliplatin-based |
| Bendell 2013^[25]^ | Multi-  center | 2008.3-2009.8 | FOLFOX+ Axitinib | FOLFOX+ Bevacizumab | 36  (16/20) | 35  (24/11) | 59  (25-75) | 60  (41-77) | ECOG:  0-1/0-1 | NA | Irinotecan-based |
| Cao 2015^[26]^ | China | 2010.6-2014.5 | FOLFIRI+ Bevacizumab | FOLFIRI | 65  (40/25) | 77  (48/29) | 62  (30–79) | 61  (24-81) | ECOG:  0-2/0-2 | NA | Oxaliplatin-based |
| Ciardiello 2016^[27]^ | Italy | 2010.2-2014.9 | FOLFOX+ Cetuximab | FOLFOX | 74  (46/28) | 79  (43/36) | 64  (35-79) | 63  (40-80) | NA/NA | WT | FOLFIRI + Cetuximab |
| Clarke 2011^[28]^ | Australia | 2005.6-2008.1 | FOLFIRI | Irinotecan | 44  (31/13) | 44  (26/18) | 64  (35–78) | 66  (26-84) | ECOG:  0-2/0-2 | NA | 5FU-based |
| Clarke 2011^[28]^ | Australia | 2005.6-2008.1 | FOLFIRI+ Ganitumab | Irinotecan | 52  (24/28) | 44  (26/18) | 58  (28–81) | 66  (26-84) | ECOG:  0-1/0-2 | NA | 5FU-based |
| Cohn 2013^[309]^ | Multi-  center | 2009.3-2011.1 | FOLFIRI+ Conatumumab | FOLFIRI | 51  (27/24) | 52  (23/29) | 59  (37–79) | 59  (32–80) | ECOG:  0-1/0-1 | MT | Oxaliplatin-based |
| Cunningham 1998^[30]^ | Multi-  center | NA | Irinotecan | Supportive Care | 189  (129/60) | 90  (52/38) | 59  (22–75) | 62  (34–75) | ECOG:  0-2/0-2 | NA | 5FU-based |
| Cunningham 2013^[31]^ | Multi-  center | 2006.1-2007.6 | FOLFOX+ Cediranib | FOLFOX+ Bevacizumab | 144  (96/48) | 66  (39/27) | NA | NA | ECOG:  0-2/0-2 | NA | Oxaliplatin-based |
| Élez 2015^[32]^ | Multi-  center | 2009.10-2010.10 | Abituzumab+ Irinotecan+ Cetuximab | Irinotecan+ Cetuximab | 145  (85/59) | 72  (45/27) | (25-83) | 58  (26-76) | ECOG:  0-1/0-1 | WT | Oxaliplatin-based |
| Eng 2016^[33]^ | Multi-  center | NA | Tivantinib+ Irinotecan+ Cetuximab | Irinotecan+ Cetuximab | 60  (26/34) | 57  (32/25) | 57(29-79) | 57  (27-79) | ECOG:  0-1/0-1 | WT | Irinotecan-based |
| Ettrich 2021^[34]^ | Multi-  center | 2012.12-2016.5 | FOLFOX+ Nintedanib | FOLFOX | 27  (17/10) | 26  (22/4) | 63  (37-78) | 65  (48-77) | ECOG:  0-1/0-1 | NA | NA |
| Giantonio 2007^[35]^ | Multi-  center | 2001.11-2003.4 | FOLFOX+ Bevacizumab | FOLFOX | 286  (113/173) | 291  (114/177) | 62  (21-85) | 61  (25-84) | ECOG:  0-2;0-2 | NA | FOLFIRI |
| Giantonio 2007^[35]^ | Multi-  center | 2001.11-2003.4 | FOLFOX+ Bevacizumab | Bevacizumab | 286  (113/173) | 243  (99/144) | 62  (21-85) | 60  (23-82) | ECOG:  0-2;0-2 | NA | FOLFIRI |
| Gibbs 2011^[36]^ | Australia | 2004.3-2006.11 | Irinotecan+ Hyaluronan | Irinotecan | 41  (24/17) | 35  (21/14) | 62  (45–75) | 63  (37–78) | ECOG:  0-1;0-1 | NA | 5FU without Oxaliplatin |
| Graeven 2007^[37]^ | Germany | 2001.9-2004.3 | FOLFIRI | Irinotecan | 28  (16/12) | 27  (20/7) | 66  (44–77) | 67  (53–78) | ECOG:  0-2;0-2 | NA | 5FU without Oxaliplatin |
| Haller 2008^[38]^ | Multi-  center | 2001.1-2004.4 | Irinotecan+ Oxaliplatin | Irinotecan | 317  (189/128) | 310  (189/121) | 62  (25-88) | 63  (28-85) | NA/NA | NA | 5FU or Capecitabine without leucovorin |
| Hecht 2017^[39]^ | Multi-  center | 2011.12-2014.3 | FOLFIRI+ Simtuzumab | FOLFIRI | 169 | 80 | (22-83) | 59  (32–85) | ECOG:  0-2;0-2 | MT | FOLFOX |
| Hecht 2015^[40]^ | America | 2006.11-2010.12 | FOLFIRI+ Panitumumab | FOLFIRI+ Bevacizumab | 91  (62/29) | 91  (58/33) | 60  (27–84) | 60  (25–80) | ECOG:  0-1;0-1 | WT | FOLFOX + Bevacizumab |
| Kim 2009^[41]^ | Multi-  center | 1999.10-2003.12 | FOLFOX | Irinotecan | 246  (135/111) | 245  (152/93) | 63  (28-83) | 63  (25-86) | ECOG:  0-2/0-2 | NA | 5FU-based |
| Lenz 2017^[42]^ | Multi-  center | 2009.2-2014.3 | Etirinotecan Pegol | Irinotecan | 42  (24/18) | 41  (25/16) | 60  (40-80) | 56  (30-83) | ECOG:  0-1/0-1 | MT  WT | 5FU-based |
| Li 2018^[43]^ | Multi-  center | 2012.7-2014.3 | FOLFIRI+ Aflibercept | FOLFIRI | 223  (128/95) | 109  (63/46) | NA | NA | ECOG:  0-1/0-1 | NA | Oxaliplatin-based |
| Liu 2015^[44]^ | China | 2010.6-2014.5 | FOLFIRI+ Bevacizumab + Panitumumab | FOLFIRI | 57  (35/22) | 69  (42/27) | (21-82) | (25-85) | ECOG:  0-2/0-2 | MT  WT | 5FU or Oxaliplatin-based |
| Moore 2016^[45]^ | Multi-  center | 2010.8-2013.10 | FOLFOX+ Ramucirumab | FOLFOX | 52  (31/21) | 49  (28/21) | NA | NA | ECOG:  0-2/0-2 | MT  WT | Irinotecan-based |
| Moore 2016^[45]^ | Multi-  center | 2010.8-2013.10 | FOLFOX+ Icrucumab | FOLFOX | 52  (23/29) | 49  (28/21) | NA | NA | ECOG:  0-1/0-2 | MT  WT | Irinotecan-based |
| Muro 2010^[46]^ | Multi-  center | 2006.1-2008.1 | Irinotecan+  S-1 | FOLFIRI | 213  (120/93) | 213  (123/93) | 61  (29–75) | 63  (32–75) | ECOG:  0-1/0-1 | NA | NA |
| O'Neil 2014^[47]^ | Multi-  center | 2009.12-2010.9 | FOLFOX+ Linifanib | FOLFOX+ Bevacizumab | 99  (56/43) | 49  (29/20) | (31-81) | 57  (22-71) | ECOG:  0-1/0-2 | NA | Irinotecan or 5FU-based |
| Peeters 2013^[48]^ | Multi-  center | 2008.12-2010.5 | FOLFIRI+ Trebananib | FOLFIRI | 95  (60/35) | 49  (24/25) | 56  (23–79) | 55  (29–79) | ECOG:  0-1/0-1 | MT  WT | FOLFOX |
| Peeters 2010^[49]^ | Multi-  center | 2006.6-2008.3 | FOLFIRI+ Panitumumab | FOLFIRI | 541  (321/220) | 542  (339/203) | (28-84) | (29-86) | ECOG:  0-2/0-2 | MT  WT | 5FU-based |
| Pietrantonio 2020^[50]^ | Italy | 2014.11-2019.5 | Capecitabine+ Temozolomide | FOLFIRI | 43  (18/25) | 43  (24/19) | 70  (63–75) | 67  (61–73) | ECOG:  0-1/0-1 | MT | Oxaliplatin-based |
| Price 2014^[51]^ | Multi-  center | 2010.2-2012.7 | Panitumumab | Cetuximab | 499  (315/184) | 500  (318/182) | 61  (54–67) | 61  (53–68) | ECOG:  0-2/0-2 | WT | FOLFOX + Bevacizumab |
| Rothenberg 2008^[52]^ | Multi-  center | 2003.7-2005.5 | XELOX | FOLFOX | 313  (194/119) | 314  (191/123) | 61  (26-81) | 60  (26-83) | ECOG:  0-2/0-2 | NA | Irinotecan-based |
| Rothenberg 2003^[53]^ | Multi-  center | 2000.11-2001.9 | FOLFOX | 5FU | 152  (87/65) | 151  (82/69) | 59  (22-88) | 60  (21-80) | NA/NA | NA | Irinotecan-based |
| Rothenberg 2003^[53]^ | Multi-  center | 2000.11-2001.9 | Oxaliplatin | 5FU | 156  (95/61) | 151  (82/69) | 61  (27-79) | 60  (21-80) | NA/NA | NA | Irinotecan-based |
| Rougier 2002^[54]^ | France | 1997.6-1998.8 | FOLFIRI | FOLFOX | 35  (23/12) | 33  (25/8) | 66  (39–76) | 65  (39–75) | ECOG:  0-2/0-2 | NA | 5FU-based |
| Rougier 2002^[54]^ | France | 1997.6-1998.8 | FOLFIRI | Irinotecan+ Oxaliplatin | 35  (23/12) | 33  (14/19) | 66  (39–76) | 60  (31–75) | ECOG:  0-2/0-2 | NA | 5FU-based |
| Rougier 1998^[55]^ | Multi-  center | 1995.10-1997.7 | Irinotecan | 5FU | 127  (72/55) | 129  (84/45) | 58  (30–75) | 58  (25–75) | ECOG:  0-2/0-2 | NA | 5FU-based regimen |
| Sakai 2020^[56]^ | Japan | 2011.12-2014.9 | Irinotecan+ Panitumumab | Irinotecan+ Cetuximab | 61  (42/19) | 59  (37/22) | 64  (41-80) | 64  (33-79) | ECOG:  0-2/0-2 | WT | FOLFORI + FOLFOX |
| Sanoff 2018^[57]^ | Multi-  center | 2011.4-2015.8 | FOLFIRI+ Regorafenib | FOLFIRI | 120  (68/52) | 61  (30/29) | 62  (30-94) | 62  (30-82) | ECOG:  0-1/0-1 | NA | FOLFOX |
| Seymour 2013^[58]^ | UK | 2006.12-2010.8 | Irinotecan+ Panitumumab | Irinotecan | 230  (160/70) | 230  (158/72) | 64  (57–70) | 63  (56-69) | ECOG:  0-2/0-2 | MT  WT | 5FU-based |
| Shah 2022^[59]^ | Multi-  center | 2016.8-2019.3 | FOLFIRI+ Napabucasin | FOLFIRI | 624  (384/240) | 629  (375/254) | 61  (26-84) | 61  (21-86) | ECOG:  0-1/0-1 | NA | FOLFOX |
| Shapiro 2018^[60]^ | Australia | 2012.11-2016.6 | Irinotecan+ Cetuximab | Cetuximab | 25  (18/7) | 21  (13/8) | 65  (38–76) | 67  (41-75) | ECOG:  0-1/0-2 | WT | FOLFOX |
| Shi 2019^[61]^ | China | 2009.5-2011.9 | Irinotecan+ CMAB009 | Irinotecan | 337  (195/142) | 164  (104/60) | 55±10.55 | 55±11.02 | ECOG:  0-1/0-2 | WT | FOLFOX |
| Shi 2017^[62]^ | China | 2011.6-2015.6 | FOLFOX+ Bevacizumab + Erlotinib | FOLFOX+ Bevacizumab | 66  (47/19) | 65  (42/23) | 63  (41-75) | 62  (38-72) | ECOG:  0-2/0-2 | NA | Xaliplatin-based or Irinotecan- based |
| Shitara 2016^[63]^ | Japan | 2011.4-2014.2 | FOLFIRI+ Panitumumab | FOLFIRI+ Bevacizumab | 59  (34/25) | 58  (39/19) | 62  (31-82) | 64  (26-78) | ECOG:  0-1/0-1 | WT | Oxaliplatin + Bevacizumab |
| Sobrero 2008^[64]^ | Multi-  center | 2003.5-2006.2 | Irinotecan+ Cetuximab | Irinotecan | 648  (405/243) | 650  (411/239) | 61  (23-85) | 62  (21-90) | ECOG:  0-2/0-2 | NA | FOLFOX |
| Tabernero 2015^[65]^ | Multi-  center | 2010.12-2013.8 | FOLFIRI+ Ramucirumab | FOLFIRI | 536  (289/247) | 536  (326/210) | 62  (21–83) | 62  (33–87) | ECOG:  0-3/0-3 | MT  WT | FOLFOX + Bevacizumab |
| Van Cutsem 2011^[66]^ | Multi-  center | 2003.1-2004.10 | FOLFOX+ Vatalanib | FOLFOX | 426  (264/162) | 429  (268/161) | 61  (21-85) | 59  (18-81) | ECOG:  0-2/0-2 | NA | FOLFIRI |
| Van Cutsem 2012^[67]^ | Multi-  center | 2007.11-2010.3 | FOLFIRI+ Aflibercept | FOLFIRI | 612  (365/247) | 614  (353/261) | 61  (21-82) | 61  (19-86) | ECOG:  0-2/0-2 | NA | Oxaliplatin-based |
| Xie 2014^[68]^ | China | 2009.12-2013.11 | FOLFIRI+ Bevacizumab+ Panitumumab | FOLFIRI | 137  (81/56) | 155  (98/57) | 61  (23-85) | 58  (21-86) | ECOG:  0-2/0-2 | NA | Oxaliplatin + 5FU-based |
| Zhang 2022^[69]^ | China | 2016.11-2020.1 | FOLFIRI | Irinotecan | 88  (52/36) | 84  (54/30) | 59  (50–66) | 61  (53–68) | ECOG:  0-2/0-2 | MT  WT | Oxaliplatin + 5FU-based |

MT: mutant type; WT: wild type; ECOG: Eastern Cooperative Oncology Group score.

Fig 1: Risk of bias of included studies


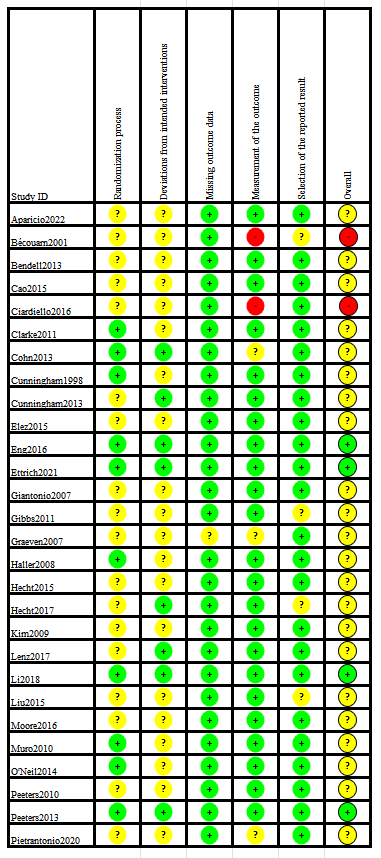

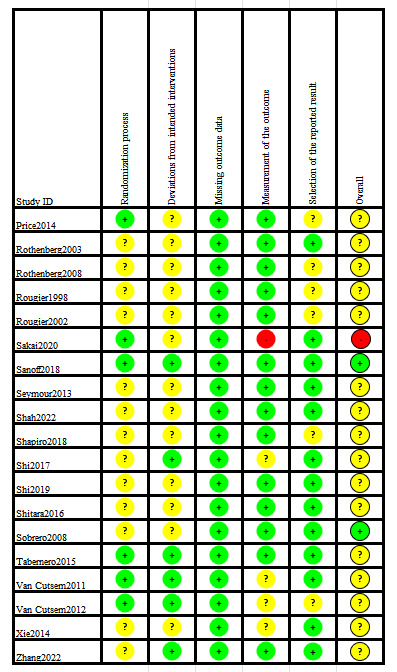

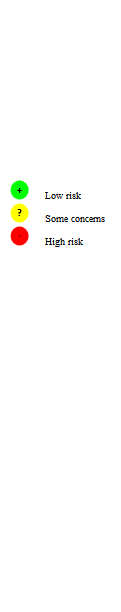


Fig 2: Risk of bias of included studies


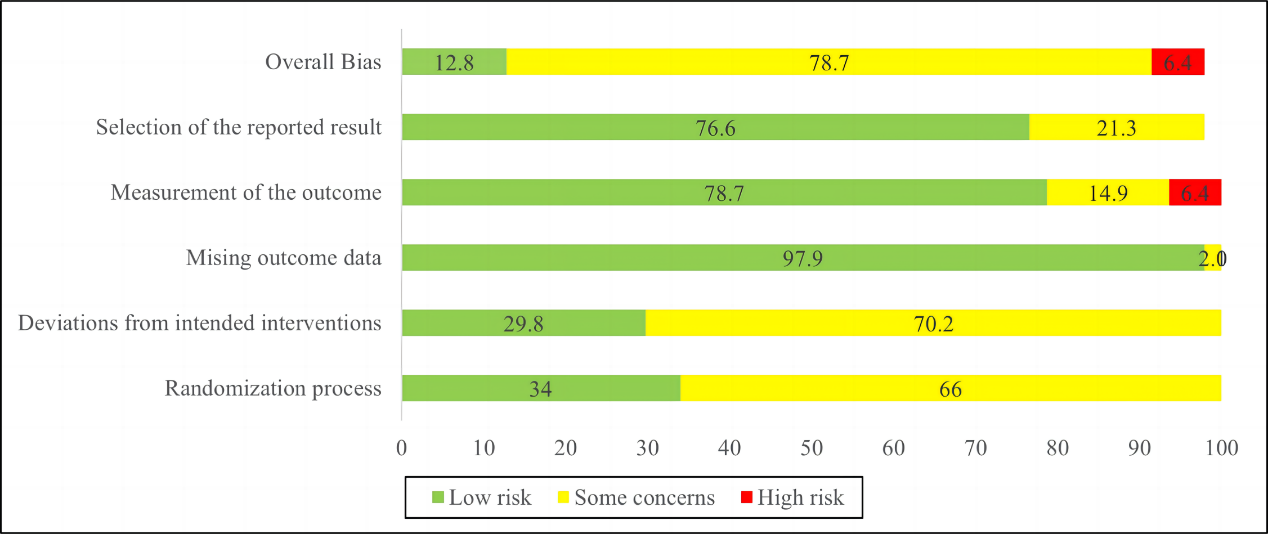


Fig 3: Inconsistency test plots of included treatments


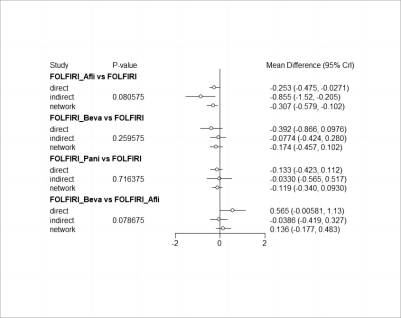

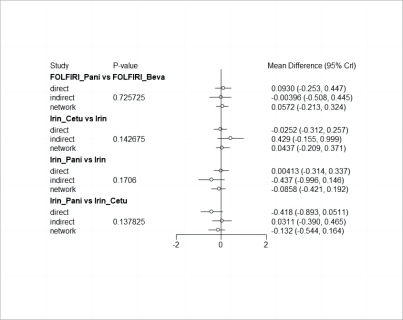


3A: Inconsistency test OS forest plot


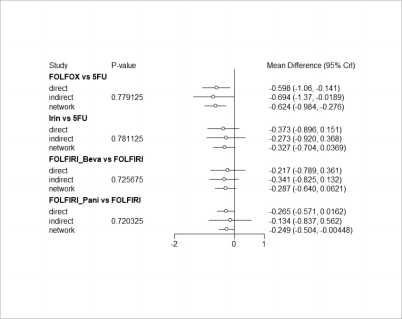

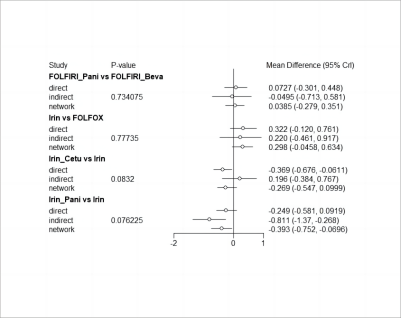

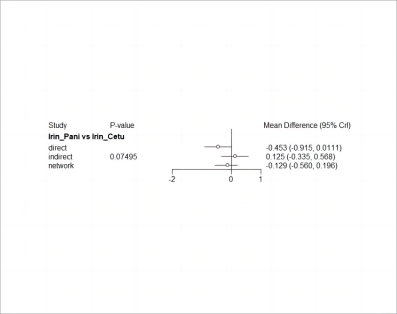


3B: Inconsistency test PFS forest plot


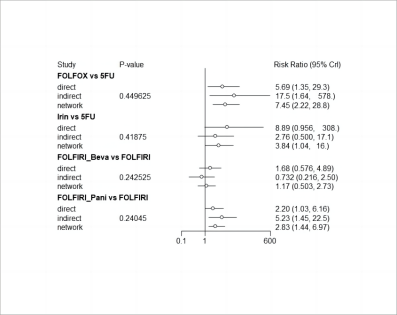

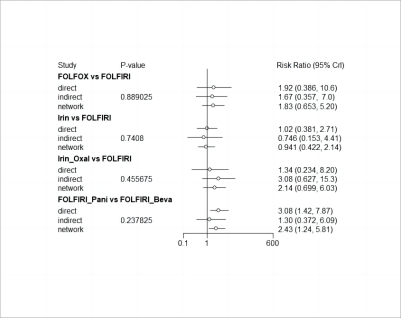

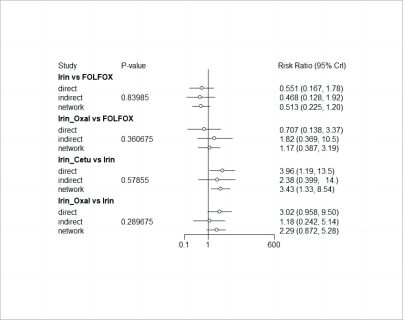

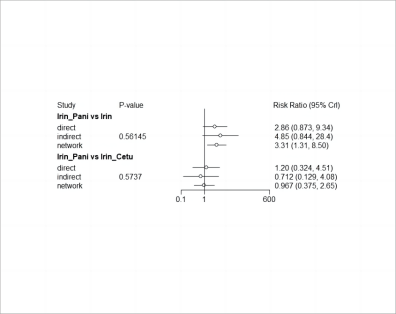


3C: Inconsistency test ORR forest plot

(continued)


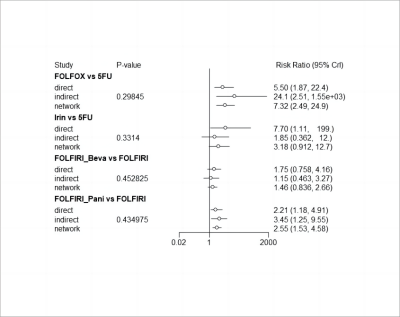

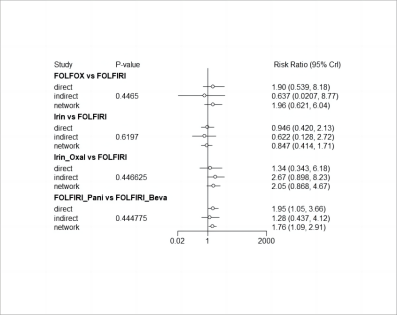

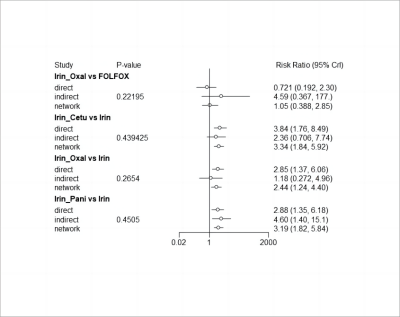


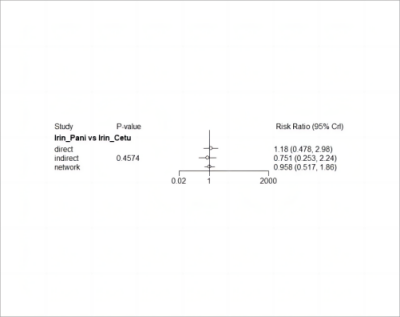


3D: Inconsistency test Partial response (PR) forest plot


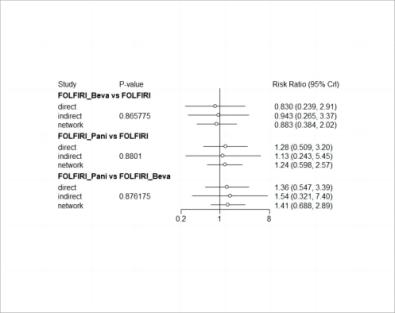


3E: Inconsistency test Grade 3 and above adverse events (Grade ≥ 3AE) forest plot


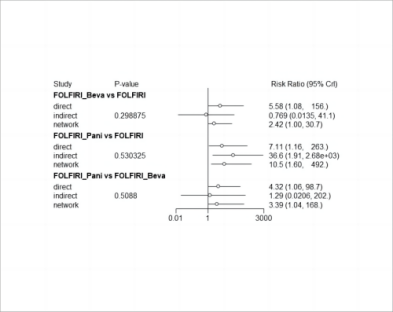


3F: Inconsistency test any adverse events (Any AE) forest plot

OS: Overall Survival; PFS: Progression-Free-Survival; ORR: Overall Response Rate; CR: Complete Response; PR: Partial Response; Grade≥3AE: Grade≥3 Adverse Events; Any AE: Any Adverse Events.

Table 3: SUCRA Probability rankings of outcomes

| Treatments | OS | PFS | ORR | CR | PR | Grade≥3AE | Any AE |
| --- | --- | --- | --- | --- | --- | --- | --- |
| 5FU | 0.12 | 0.11 | 0.07 | 0.55 | 0.07 | 0.69 |  |
| Abit_Irin_Cetu | 0.56 | 0.42 | 0.70 |  |  | 0.36 | 0.29 |
| Beva | 0.43 | 0.29 | 0.23 |  | 0.28 | 0.66 |  |
| Cape_Temo | 0.51 | 0.23 | 0.35 |  | 0.21 | 0.92 | 0.91 |
| Cetu | 0.13 | 0.20 | 0.27 | 0.72 | 0.26 | 0.69 |  |
| EP | 0.50 | 0.66 | 0.55 |  | 0.55 | 0.57 | 0.73 |
| FOLFIRI | 0.47 | 0.33 | 0.31 | 0.38 | 0.31 | 0.64 | 0.76 |
| FOLFIRI_Afli | 0.82 | 0.66 | 0.65 | 0.26 | 0.65 | 0.46 | 0.64 |
| FOLFIRI_Axit | 0.39 | 0.40 | 0.40 | 0.46 | 0.48 | 0.35 | 0.41 |
| FOLFIRI_Beva | 0.68 | 0.60 | 0.38 | 0.46 | 0.46 | 0.69 |  |
| FOLFIRI_Beva_Pani | 0.83 | 0.62 | 0.46 | 0.37 | 0.48 | 0.59 |  |
| FOLFIRI_Cona | 0.60 | 0.65 | 0.87 |  | 0.89 | 0.42 | 0.75 |
| FOLFIRI_FOLFOX |  |  | 0.20 |  |  |  |  |
| FOLFIRI_Gani | 0.29 | 0.35 | 0.76 |  | 0.77 | 0.55 | 0.81 |
| FOLFIRI_Napa | 0.51 | 0.31 | 0.31 |  |  | 0.57 | 0.70 |
| FOLFIRI_Pani | 0.62 | 0.56 | 0.69 | 0.45 | 0.68 | 0.51 | 0.19 |
| FOLFIRI_Ramu | 0.68 | 0.55 | 0.35 | 0.26 | 0.36 | 0.51 |  |
| FOLFIRI_Rego | 0.48 | 0.62 | 0.47 |  | 0.48 | 0.48 |  |
| FOLFIRI_Simt | 0.21 | 0.14 | 0.31 |  | 0.31 |  |  |
| FOLFIRI_Treb | 0.59 | 0.22 | 0.88 | 0.44 | 0.86 | 0.64 | 0.75 |
| FOLFOX | 0.48 | 0.55 | 0.52 |  | 0.57 | 0.43 |  |
| FOLFOX_Axit | 0.89 | 0.74 | 0.77 |  | 0.82 | 0.18 |  |
| FOLFOX_Beva | 0.74 | 0.83 | 0.81 |  | 0.86 | 0.35 |  |
| FOLFOX_Beva_Erlo | 0.93 | 0.71 | 0.70 |  |  |  |  |
| FOLFOX_Cedi | 0.56 | 0.65 | 0.69 |  | 0.76 | 0.35 |  |
| FOLFOX_Cetu | 0.64 | 0.73 | 0.17 |  | 0.73 |  |  |
| FOLFOX_Icru | 0.31 | 0.21 | 0.78 |  | 0.21 | 0.37 |  |
| FOLFOX_Lini | 0.39 | 0.56 | 0.50 |  |  | 0.29 |  |
| FOLFOX_Nint | 0.48 | 0.82 | 0.17 |  | 0.52 | 0.31 |  |
| FOLFOX_Ramu | 0.34 | 0.46 | 0.70 |  | 0.21 | 0.45 |  |
| FOLFOX_Vata | 0.48 | 0.72 |  |  |  | 0.37 |  |
| Irin | 0.37 | 0.28 | 0.29 | 0.36 | 0.27 | 0.58 | 0.36 |
| Irin_Cetu | 0.32 | 0.51 | 0.71 | 0.75 | 0.71 | 0.40 | 0.31 |
| Irin_CMAB009 |  | 0.86 | 0.63 | 0.54 | 0.63 | 0.38 | 0.25 |
| Irin_Hyal | 0.58 | 0.73 | 0.61 |  | 0.63 | 0.41 |  |
| Irin_Oxal | 0.67 | 0.75 | 0.58 | 0.71 | 0.60 | 0.47 |  |
| Irin_Pani | 0.49 | 0.65 | 0.70 | 0.59 | 0.70 | 0.37 | 0.07 |
| Irin_S1 | 0.59 | 0.28 | 0.37 | 0.40 | 0.28 |  |  |
| Oxal |  | 0.08 | 0.13 |  | 0.13 | 0.62 |  |
| Pani | 0.16 | 0.20 | 0.31 | 0.80 | 0.30 | 0.66 |  |
| SupportiveCare | 0.06 | 0.65 |  |  |  | 0.65 |  |
| Tiva_Irin_Cetu | 0.69 | 0.58 | 0.78 |  | 0.80 |  | 0.07 |
| XELOX | 0.45 | 0.35 | 0.56 |  |  | 0.54 |  |

OS: Overall Survival; PFS: Progression-Free-Survival; ORR: Overall Response Rate; CR: Complete Response; PR: Partial Response; Grade≥3AE: Grade≥3 Adverse Events; Any AE: Any Adverse Events; Abit_Irin_Cetu: Abituzumab + Irinotecan+ Cetuximab; Beva: Bevacizumab; Cape_Temo: Capecitabine + Temozolomide; Cetu: Cetuximab; EP: Etirinotecan Pegol; FOLFIRI_Afli: FOLFIRI + Aflibercept; FOLFIRI_Axit: FOLFIRI+Axitinib; FOLFIRI_Beva: FOLFIRI + Bevacizumab; FOLFIRI_Beva_Pani: FOLFIRI + Bevacizumab + Panitumumab; FOLFIRI_Cona: FOLFIRI + Conatumumab; FOLFIRI_FOLFOX: FOLFIRI + FOLFOX; FOLFIRI_Gani: FOLFIRI + Ganitumab; FOLFIRI_Napa: FOLFIRI + Napabucasin; FOLFIRI_Pani: FOLFIRI + Panitumumab; FOLFIRI_Ramu: FOLFIRI + Ramucirumab; FOLFIRI_Rego: FOLFIRI + Regorafenib; FOLFIRI_Simt: FOLFIRI + Simtuzumab; FOLFIRI_Treb: FOLFIRI + Trebananib; FOLFOX_Axit: FOLFOX + Axitinib; FOLFOX_Beva: FOLFOX + Bevacizumab; FOLFOX_Beva_Erlo: FOLFOX + Bevacizumab + Erlotinib; FOLFOX_Cedi: FOLFOX + Cediranib; FOLFOX_Cetu: FOLFOX + Cetuximab; FOLFOX_Icru: FOLFOX + Icrucumab; FOLFOX_Lini: FOLFOX + Linifanib; FOLFOX_Nint: FOLFOX + Nintedanib; FOLFOX_Ramu: FOLFOX + Ramucirumab; FOLFOX_Vata: FOLFOX + Vatalanib; Irin: Irinotecan; Irin_Cetu: Irinotecan + Cetuximab; Irin_CMAB009: Irinotecan + CMAB009; Irin_Hyal: Irinotecan + Hyaluronan; Irin_Oxal: Irinotecan + Oxaliplatin; Irin_Pani: Irinotecan + Panitumumab; Irin_S1: Irinotecan + S-1; Oxal: Oxaliplatin; Pani: Panitumumab; SupportiveCare: Supportive Care; Tiva_Irin_Cetu: Tivantinib + Irinotecan + Cetuximab

Table 4: SUCRA probability rankings of OS and PFS in subgroup analyses.

|  | MT RAS | | WT RAS | |
| --- | --- | --- | --- | --- |
| Treatments | OS | PFS | OS | PFS |
| Abit_Irin_Cetu |  |  | 0.47 | 0.34 |
| Cape_Temo | 0.51 |  |  |  |
| Cetu |  |  | 0.13 | 0.18 |
| EP | 0.56 | 0.75 |  |  |
| FOLFIRI | 0.52 | 0.41 | 0.43 | 0.33 |
| FOLFIRI_Beva |  |  | 0.73 | 0.65 |
| FOLFIRI_Beva_Pani | 0.88 | 0.7 | 0.66 | 0.83 |
| FOLFIRI_Cona | 0.57 | 0.67 |  |  |
| FOLFIRI_Gani | 0.34 | 0.42 |  |  |
| FOLFIRI_Pani | 0.54 | 0.54 | 0.64 | 0.61 |
| FOLFIRI_Ramu | 0.57 | 0.56 | 0.63 | 0.54 |
| FOLFIRI_Simt | 0.29 | 0.23 |  |  |
| FOLFIRI_Treb | 0.47 | 0.13 | 0.58 | 0.4 |
| Irin | 0.5 | 0.52 | 0.53 | 0.43 |
| Irin_Cetu |  |  | 0.3 | 0.41 |
| Irin_CMAB009 |  |  | 0.52 | 0.85 |
| Irin_Pani | 0.28 | 0.74 | 0.63 | 0.71 |
| Pani |  |  | 0.17 | 0.19 |
| Tiva_Irin_Cetu |  |  | 0.58 | 0.53 |

MT: mutant type; WT: wild type; OS: Overall Survival; PFS: Progression-Free-Survival; Abit_Irin_Cetu：Abituzumab + Irinotecan+ Cetuximab; Cape_Temo: Capecitabine + Temozolomide; Cetu: Cetuximab; EP: Etirinotecan Pegol; FOLFIRI_ Beva: FOLFIRI + Bevacizumab; FOLFIRI_Beva_Pani: FOLFIRI + Bevacizumab + Panitumumab; FOLFIRI_Cona: FOLFIRI + Conatumumab; FOLFIRI_Cona: FOLFIRI + Conatumumab; FOLFIRI_Cona: FOLFIRI + Conatumumab; FOLFIRI_Pani: FOLFIRI + Panitumumab; FOLFIRI_Ramu: FOLFIRI + Ramucirumab; FOLFIRI_Simt: FOLFIRI + Simtuzumab; FOLFIRI_Treb: FOLFIRI + Trebananib; Irin: Irinotecan; Irin_Cetu: Irinotecan + Cetuximab; Irin_CMAB009: Irinotecan + CMAB009; Irin_Pani: Irinotecan + Panitumumab; Pani: Panitumumab; Tiva_Irin_Cetu: Tivantinib + Irinotecan + Cetuximab

Fig 4: Indirect comparison diagrams of included treatments


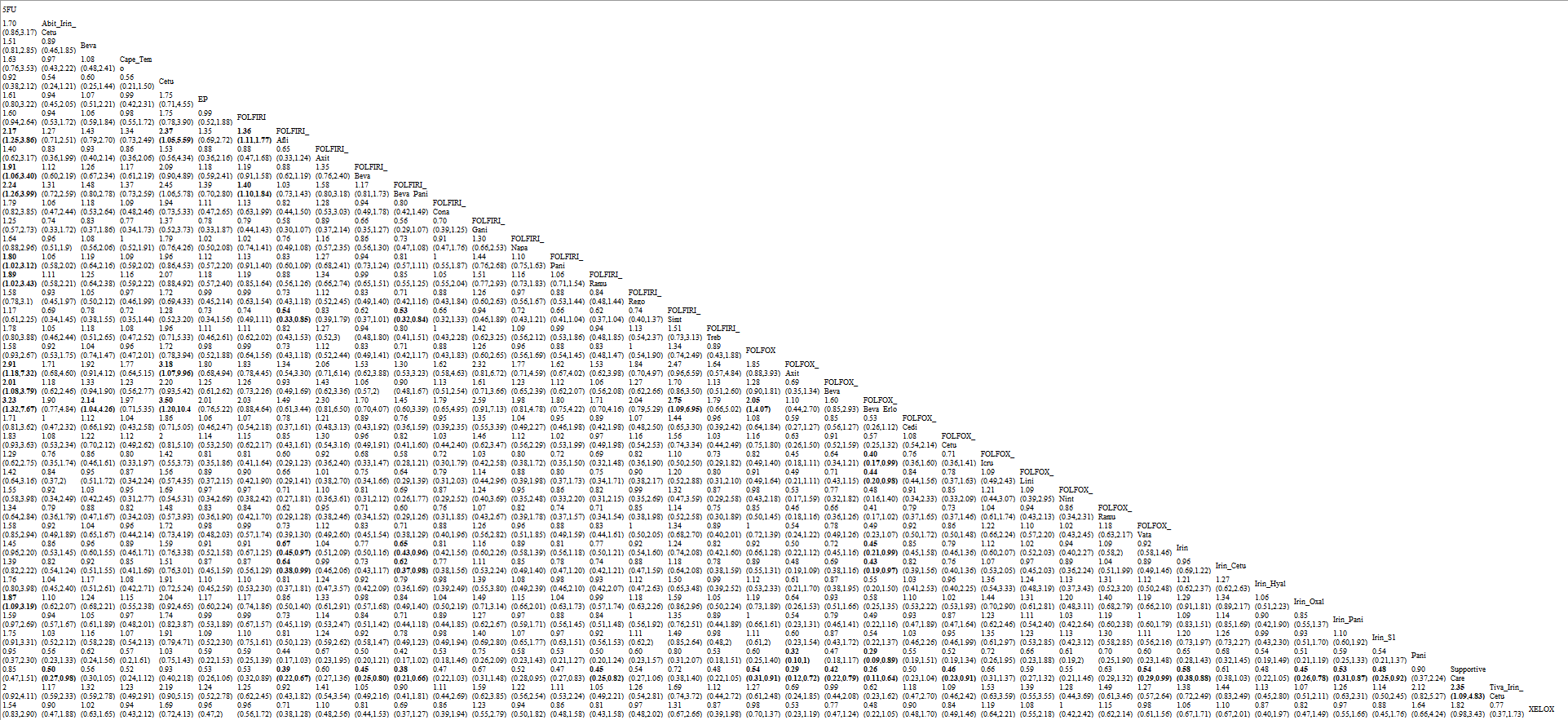


4A: Indirect comparison diagrams of OS


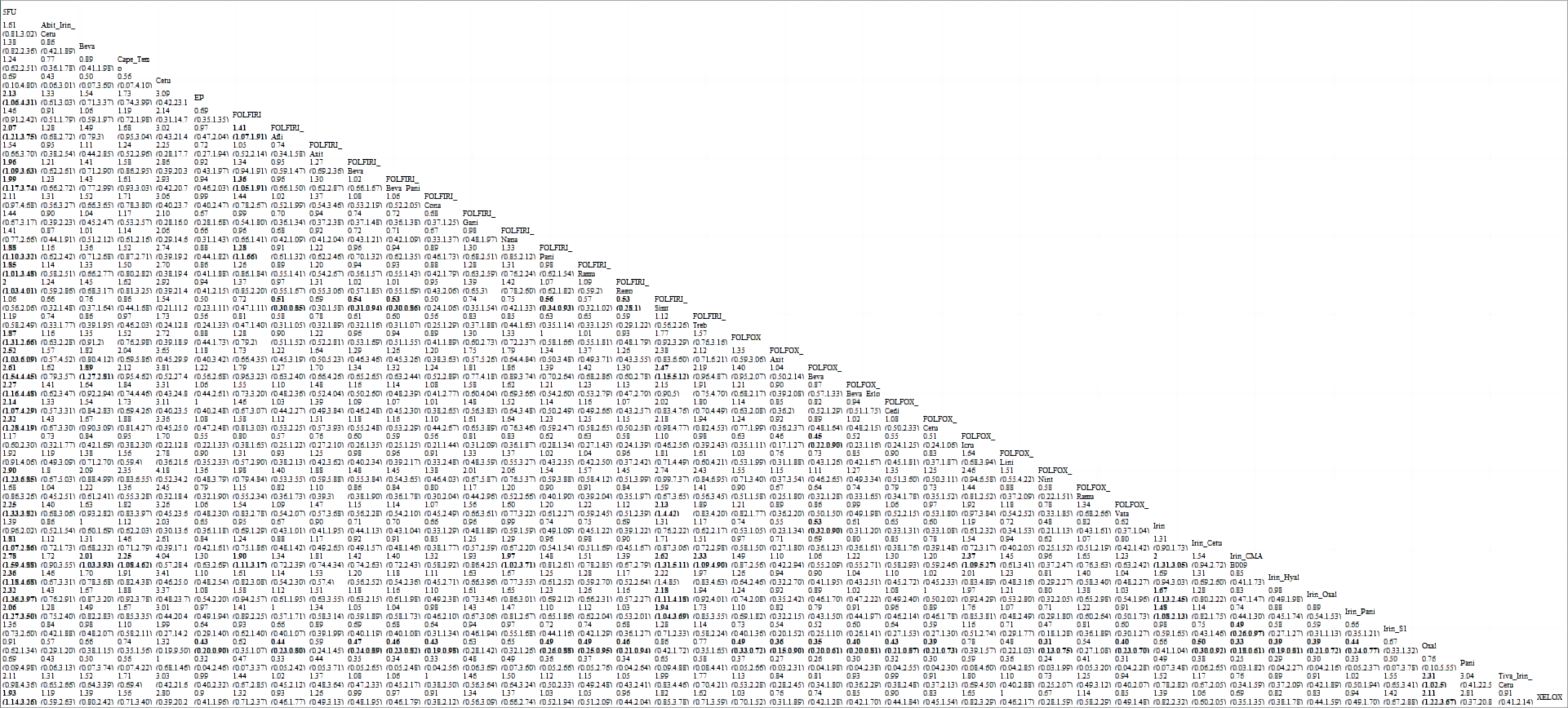
4B: Indirect comparison diagrams of PFS


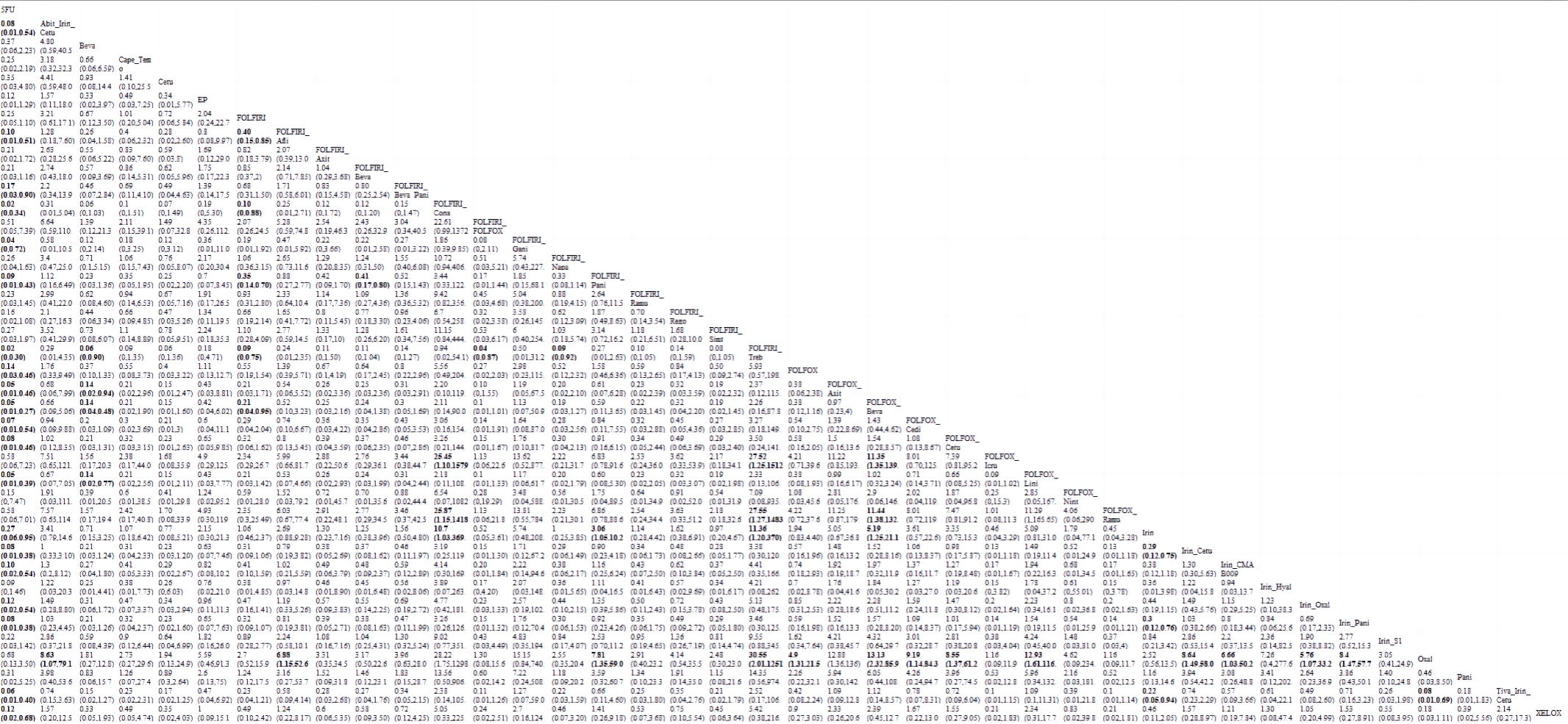


4C: Indirect comparison diagrams of ORR


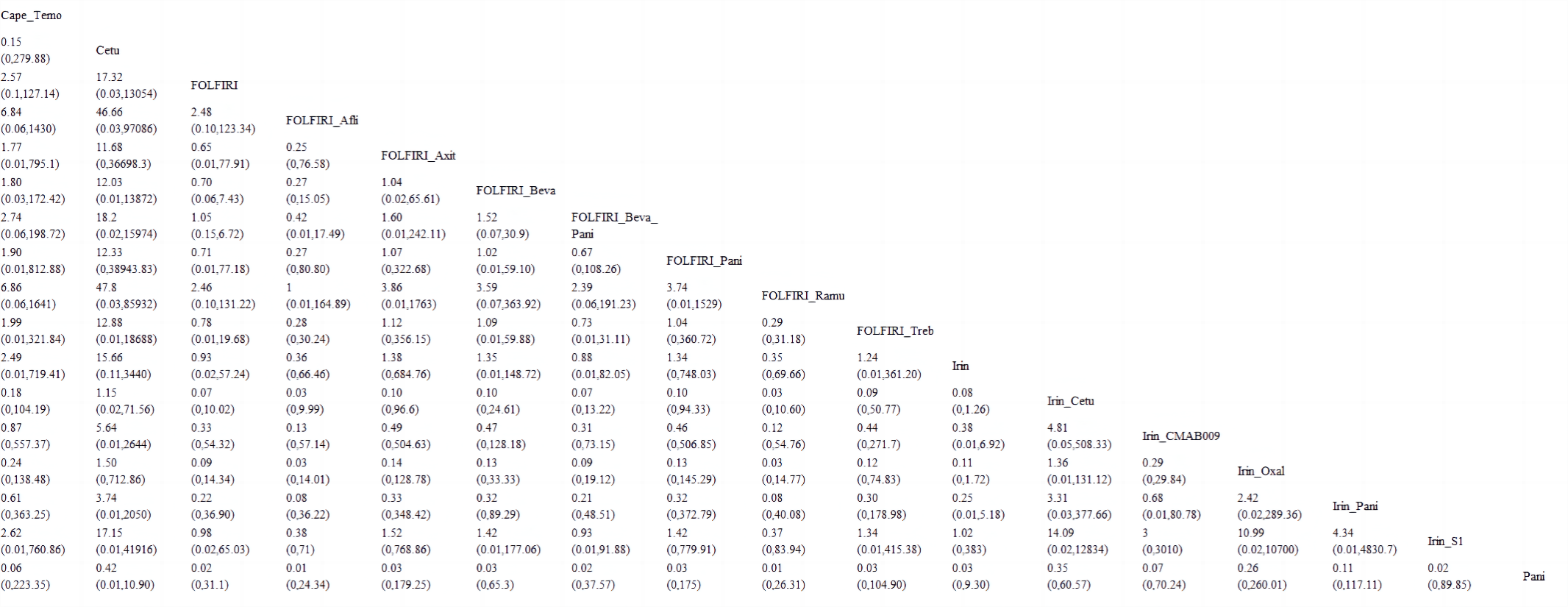


4D: Indirect comparison diagrams of CR


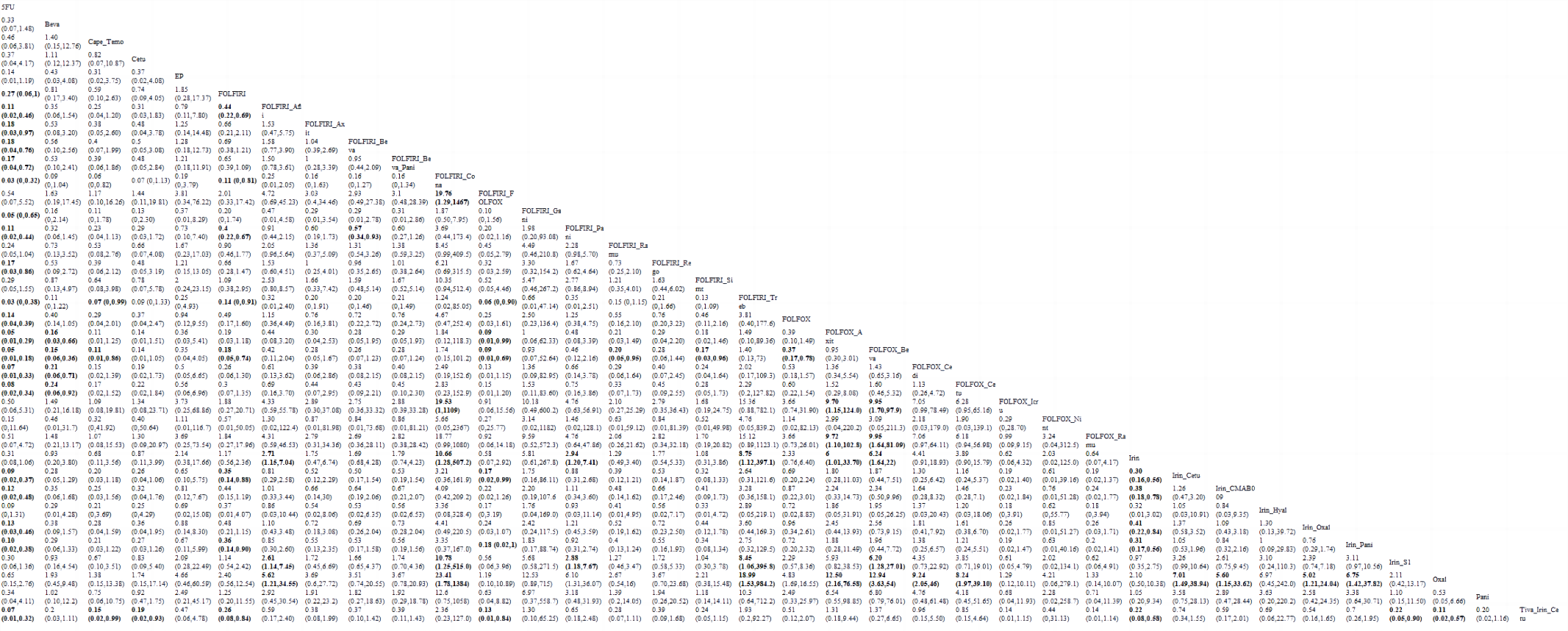


4E: Indirect comparison diagrams of PR


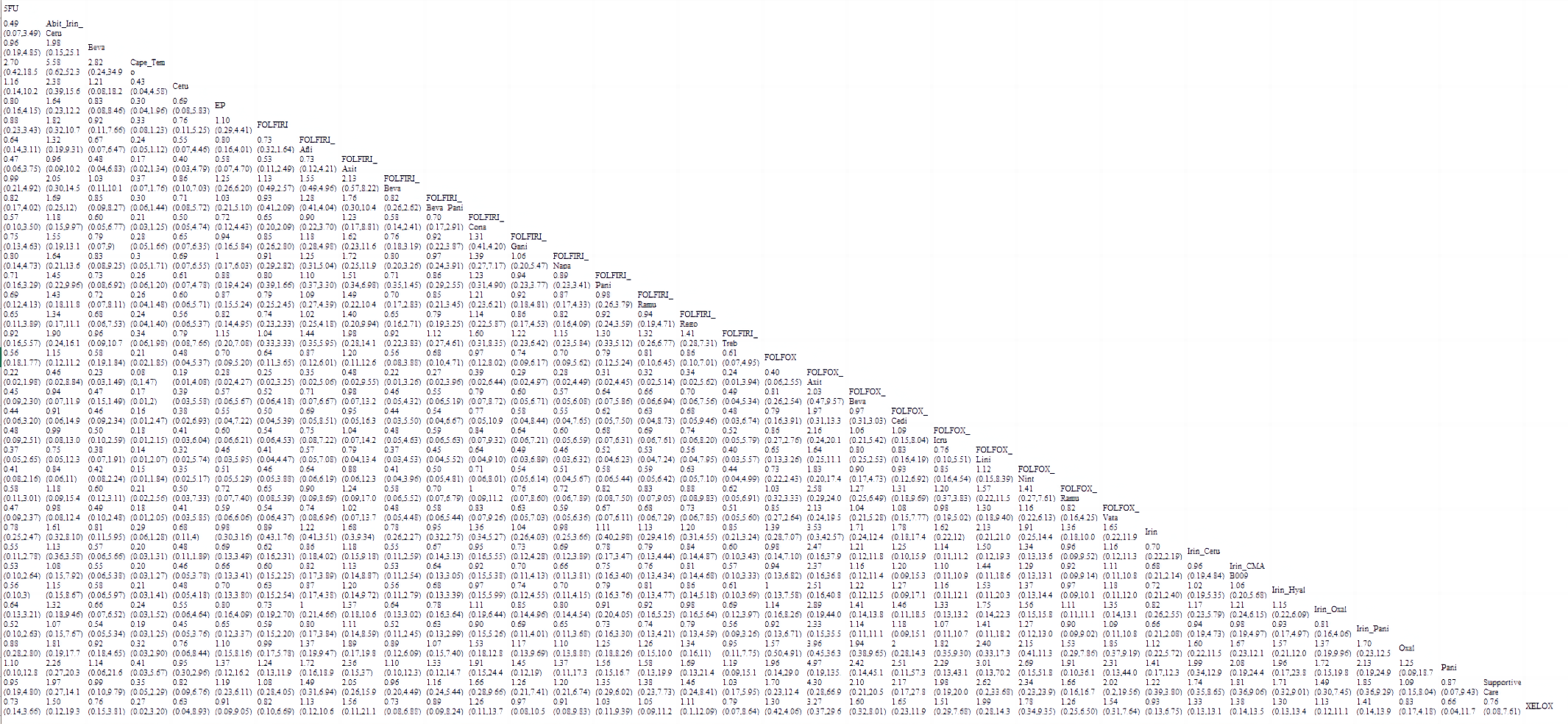


4F: Indirect comparison diagrams of Grade≥3AE


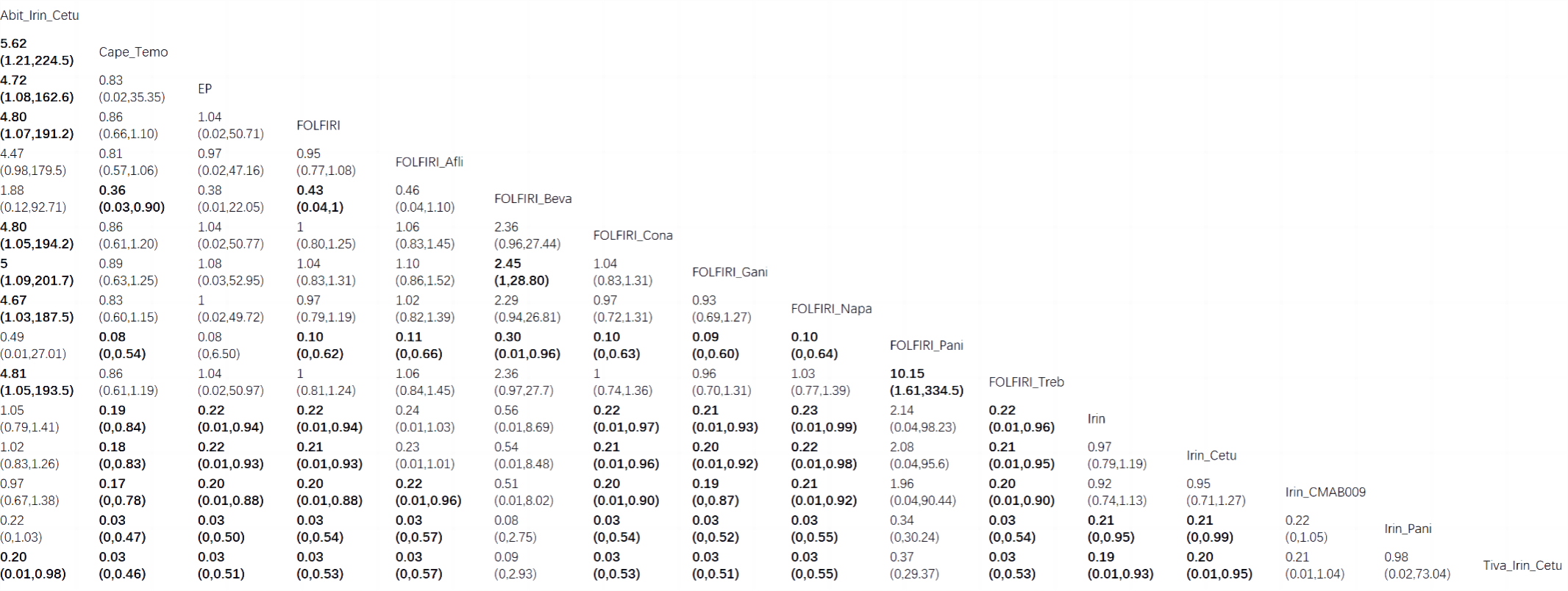


4G: Indirect comparison diagrams of Any AE

OS: Overall Survival: PFS: Progression-Free-Survival; ORR: Overall Response Rate; CR: Complete Response; PR: Partial Response; Grade≥3AE: Grade≥3 Adverse Events; Any AE: Any Adverse Events

.

Fig 5: Sensitivity analysis plots of available evidence


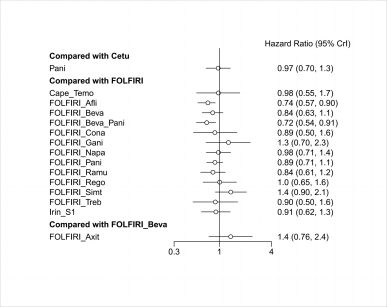

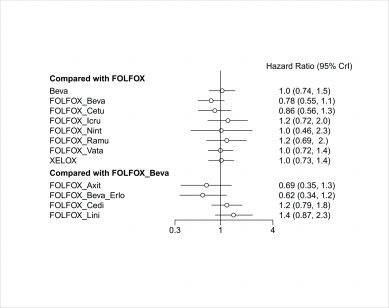

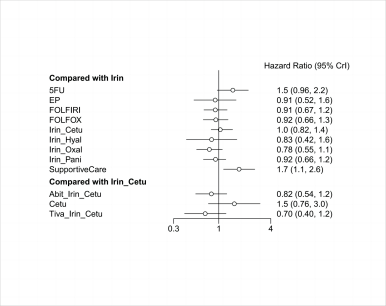


5A: Sensitivity analysis plots of OS


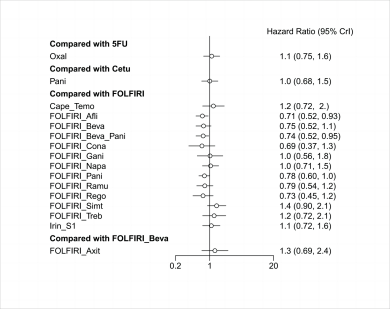

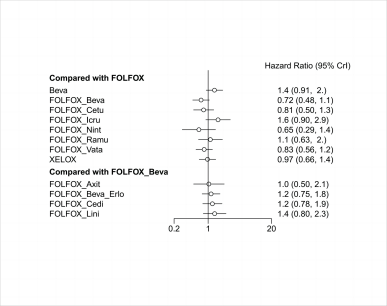

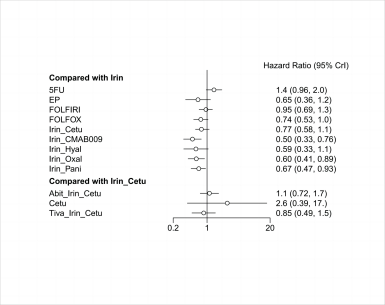


5B: Sensitivity analysis plots of PFS


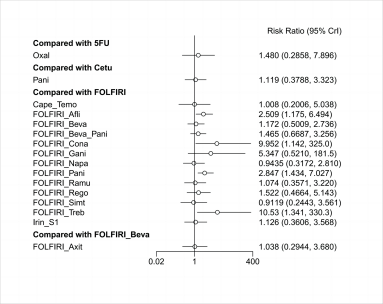

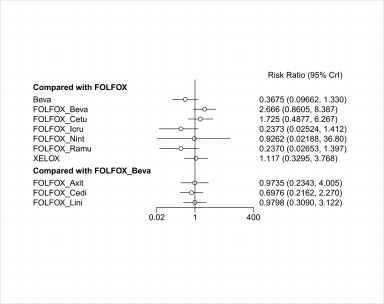

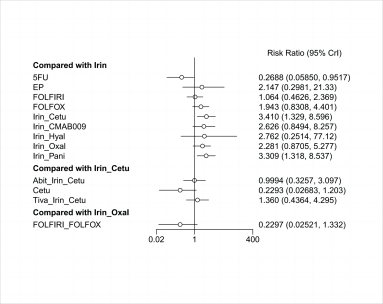


5C: Sensitivity analysis plots of ORR


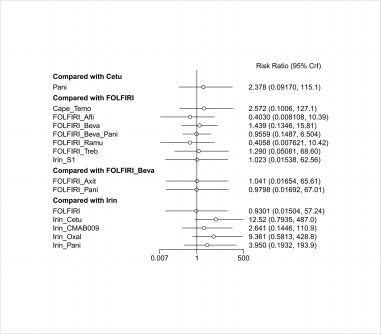

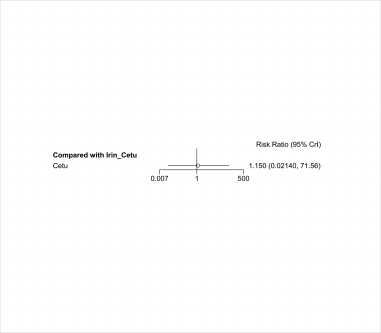


5D: Sensitivity analysis plots of CR


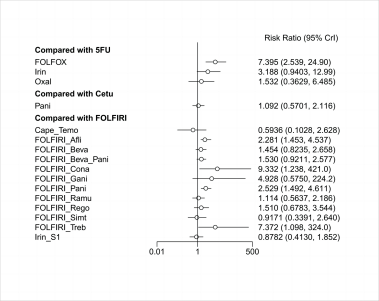

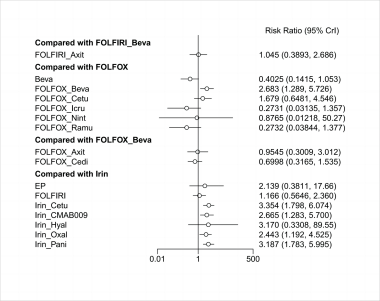

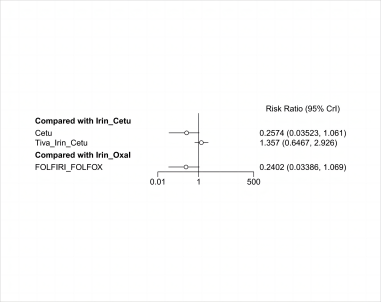


5E: Sensitivity analysis plots of PR

（continued）


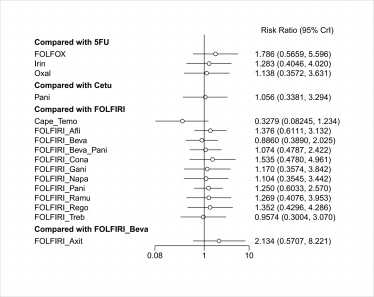

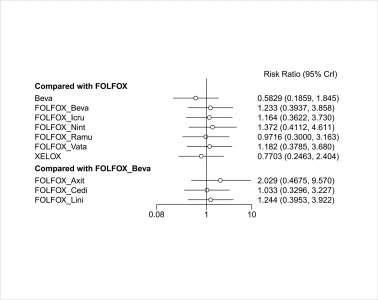

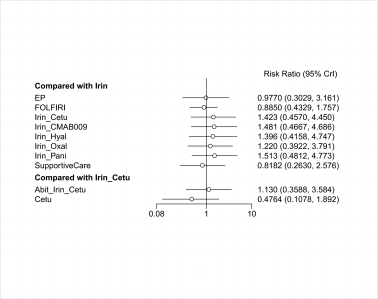


5F: Sensitivity analysis plots of Grade≥3AE


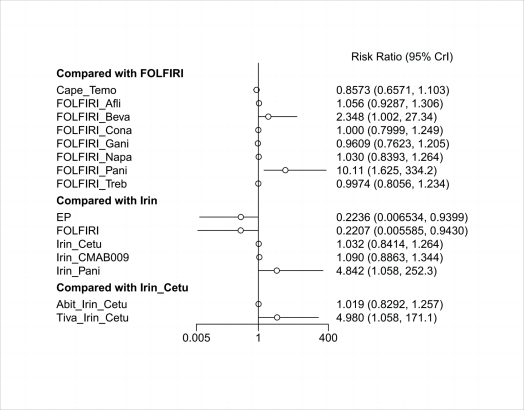


5G: Sensitivity analysis plots of Any AE

OS: Overall Survival; PFS: Progression-Free-Survival; ORR: Overall Response Rate; CR: Complete Response; PR: Partial Response; Grade≥3AE: Grade≥3 Adverse Events; Any AE: Any Adverse Events.

Fig 6: Funnel plots of included treatments


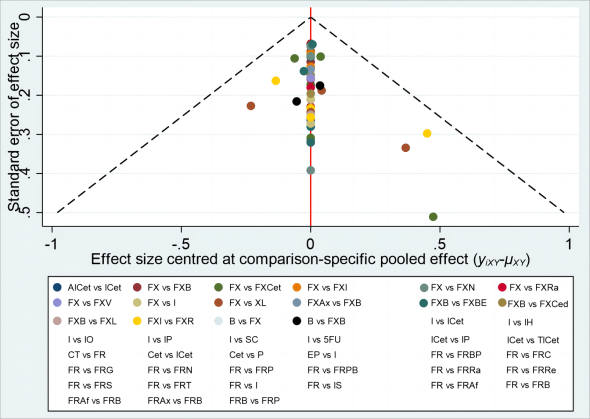

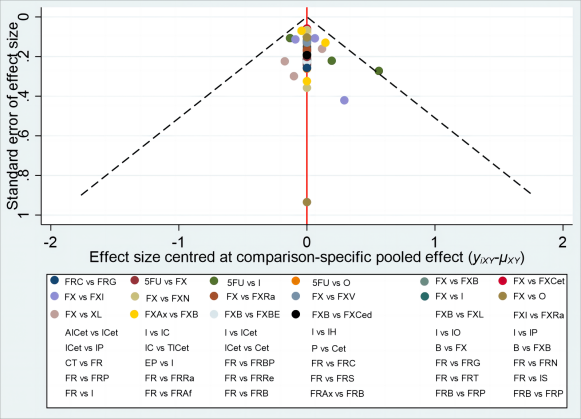


6A: Funnel plots of OS 6B:Funnel plots of PFS


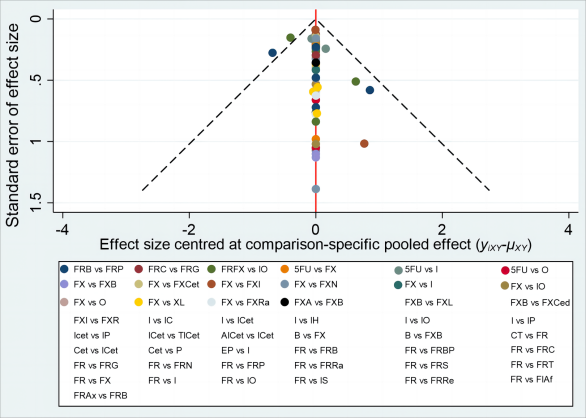

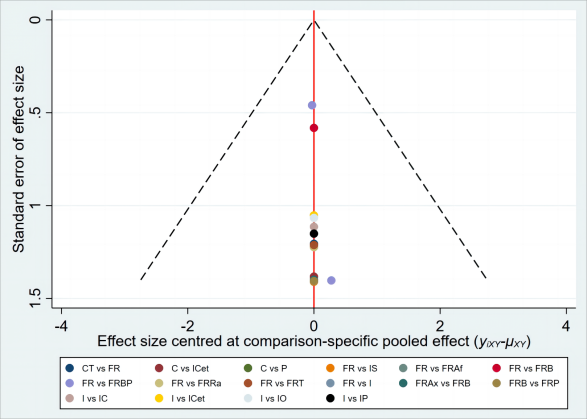


6C:Funnel plots of ORR 6D:Funnel plots of CR


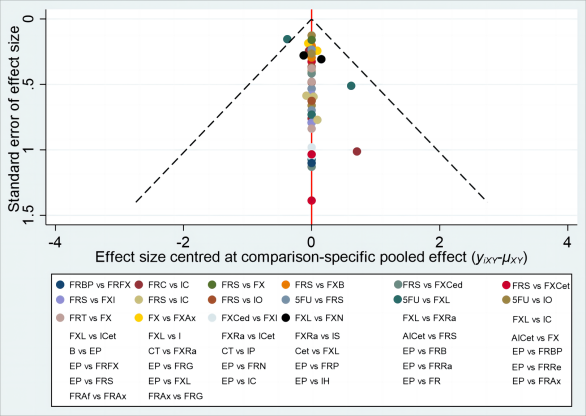

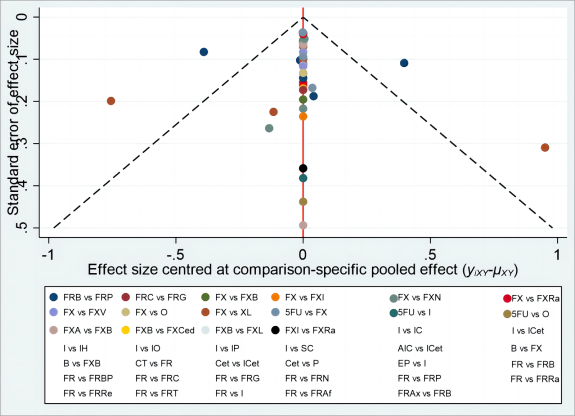


6E:Funnel plots of PR 6F:Funnel plots of Grade≥3AE


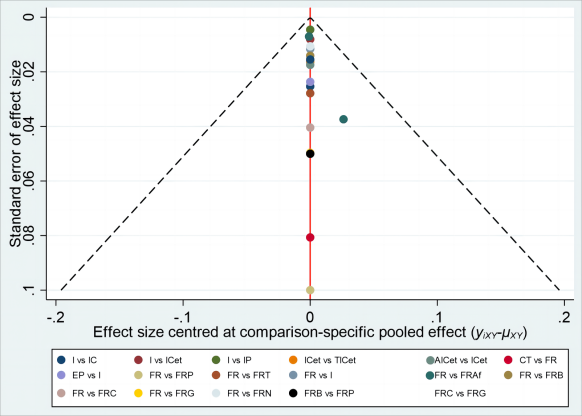


6G:Funnel plots of Any AE

OS: Overall Survival; PFS: Progression-Free-Survival; ORR: Overall Response Rate; CR: Complete Response; PR: Partial Response; Grade≥3AE:Grade≥3 Adverse Events; Any AE: Any Adverse Events; AIC, Abituzumab + Irinotecan + Cetuximab; B, Bevacizumab; CT, Capecitabine + Temozolomide; Cet, Cetuximab; EP, Etirinotecan Pegol; FR,FOLFIRI; FRAf, FOLFIRI + Aflibercept; FRAx, FOLFIRI + Axitinib;F RB, FOLFIRI + Bevacizumab; FRBP, FOLFIRI + Bevacizumab + Panitumumab; FRC: FOLFIRI + Conatumumab; FRG: FRFX: FOLFIRI + FOLFOX;FOLFIRI + Ganitumab; FRN: FOLFIRI + Napabucasin; FRP: FOLFIRI + Panitumumab; FRRa: FOLFIRI + Ramucirumab; FRRe: FOLFIRI + Regorafenib; FRT: FRS: FOLFIRI + Simtuzumab; FOLFIRI + Trebananib; FX:FOLFOX; FXA: FOLFIRI + Axitinib; FXB: FOLFOX + Bevacizumab; FXBE: FOLFOX + Bevacizumab + Erlotinib; FXCed: FOLFOX + Cediranib; FXCet: FOLFOX + Cetuximab; FXI: FOLFOX + Icrucumab; FXL: FOLFOX + Linifanib; FXN: FOLFOX + Nintedanib; FXRa: FOLFOX + Ramucirumab; FXV: FOLFOX + Vatalanib; I: Irinotecan; IC: Irinotecan + CMAB009;ICet: Irinotecan + Cetuximab; IH: Irinotecan + Hyaluronan; IO: Irinotecan + Oxaliplatin; IP: Irinotecan + Panitumumab;IS1: Irinotecan + S-1;TICet: Tivantinib + Irinotecan + Cetuximab; O: Oxaliplatin; P: Panitumumab; SC: Supportive Care; XL:XELOX

Fig 7: Subgroup analysis of SUCRA diagrams of OS and PFS.


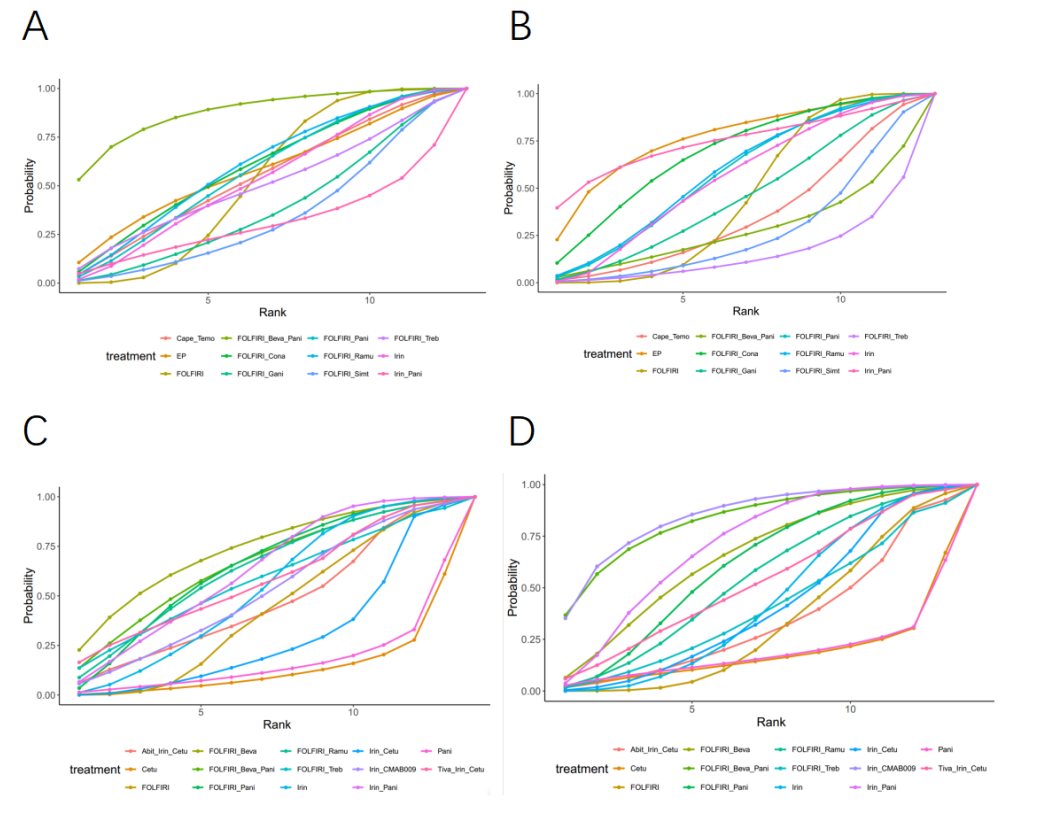


(A): SUCRA diagrams of OS in RAS mutant-type populations; (B): SUCRA diagrams of PFS in RAS mutant-type populations; (C): SUCRA diagrams OS in RAS wild-type populations; (D): SUCRA diagrams of PFS in RAS wild-type populations.

Fig 8: Subgroup analysis of indirect comparison diagrams of included treatments.


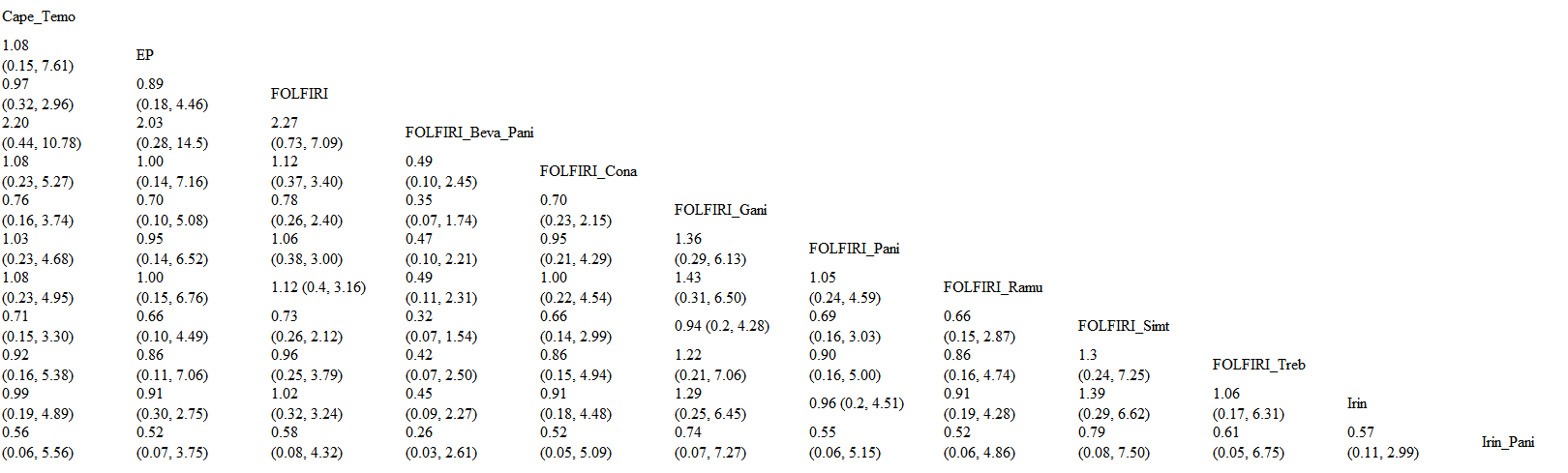


8A: Indirect comparison diagrams of OS in RAS mutant populations.


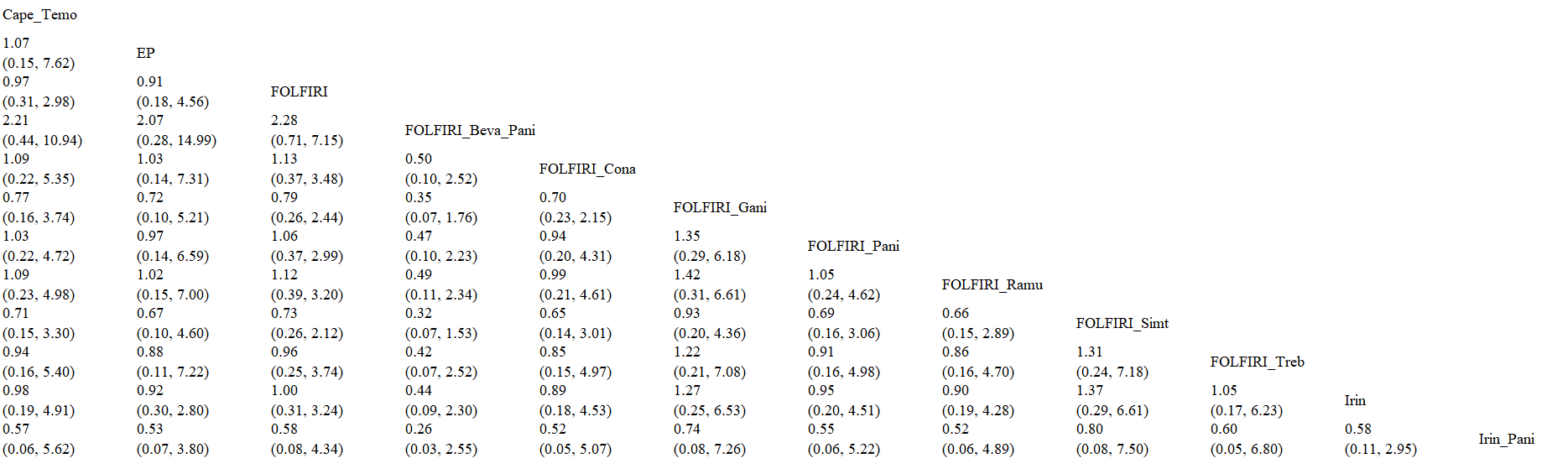


8B: Indirect comparison diagrams of PFS in RAS mutant populations.


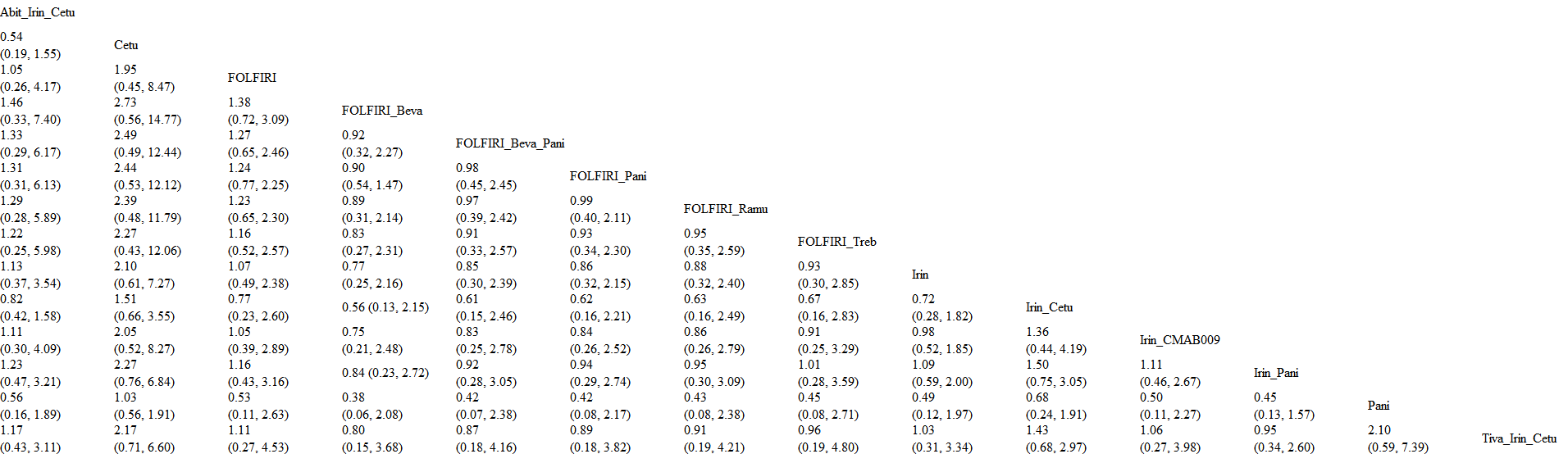


8C: Indirect comparison diagrams of OS in RAS wild-type populations.


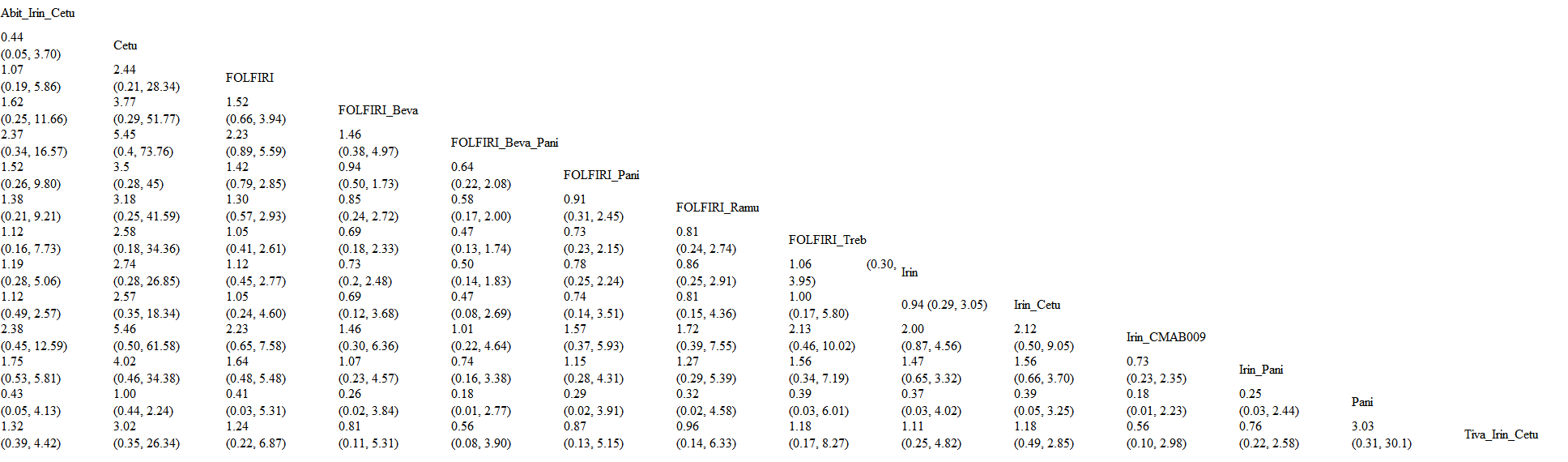


8D: Indirect comparison diagrams of PFS in RAS wild-type populations.

OS: Overall Survival: PFS: Progression-Free-Survival.
